# Strong and weak environmental perturbations cause contrasting restructure of ant transportation networks

**DOI:** 10.1098/rspb.2024.2342

**Published:** 2025-04-09

**Authors:** Imre Sándor Piross, Valentin Lecheval, Scott Powell, Matina C. Donaldson-Matasci, Elva J. H. Robinson

**Affiliations:** ^1^ Department of Biology, University of York, York, UK; ^2^ HUN-REN Centre for Ecological Research, Institute of Ecology and Botany, Budapest, Hungary; ^3^ Department of Biology, Faculty of Life Sciences, Institute for Theoretical Biology, Humboldt-Universität zu Berlin, Berlin, Germany; ^4^ Science of Intelligence, Research Cluster of Excellence, Berlin, Germany; ^5^ Department of Biological Sciences, The George Washington University, Washington, DC, USA; ^6^ Department of Biology, Harvey Mudd College, Claremont, CA, USA

**Keywords:** resilience, resistance, polydomy, network shocks, *Formica*

## Abstract

Dynamic transportation networks are embedded in all levels of biological organization. Ever-growing anthropogenic disturbances and an increasingly variable climate highlight the importance of understanding how these networks restructure under environmental perturbations. Polydomous wood ants provide a convenient model system to study the resilience of self-organizing multi-source, multi-sink transportation networks. We used 10 years of longitudinal empirical data on both unperturbed and experimentally manipulated colony networks to develop and validate a comprehensive dynamic simulation model to study network restructuring after resource removal. We performed simulation experiments to study the effects of excluding food sources with varying importance, either temporarily or permanently, imitating pulse and press perturbations of the networks. We found that removing heavily used resources, corresponding to a strong targeted perturbation, persistently decreased network efficiency, unlike random or weak perturbations. We also found that strong perturbations had excessively adverse effects on robustness and function, reducing the networks’ ability to withstand potential future perturbations. When transportation networks develop around the efficient use of a few key resources, they may be unable to quickly recover from the loss of these through self-organized restructuring. Our findings highlight the importance of considering the interaction of perturbation strength and network structure in studying transportation network dynamics.

## 1. Introduction

Resource exchange is a fundamental mode of cooperation in biological systems [1]. When multiple actors engage in the exchange of resources, they form transportation networks [2]. The emergence of these networks brings profound changes for its members, who are now exposed to the success or failure of other actors. The collective impacts of these interactions rippling through the network create their own emergent properties. Studying biological systems from a network perspective generates insight into how cells transport organelles and communicate [3], how the topology of blood vessels contributes to a robust supply of oxygen to the brain [4] and how ecologically important fungi develop their vast yet efficient fluid transport networks [5]. While such networks provide critical infrastructure within individual organisms, they also exist in complex societies, or ‘superorganisms’. Polydomy [6] refers to the phenomenon in which a single ant colony uses multiple nests to store food and rear offspring. Each polydomous ant colony can then form an intricate trail network, used to efficiently transport food, brood and other essential resources between the various nests [7,8]. These polydomous ant colony transport networks are often highly conspicuous, offering especially tractable properties for addressing general knowledge gaps in how biological networks are optimized. All biological transportation networks, ranging in scale from subcellular to whole communities, share the same crucial optimization problem [9].

The archetypical optimization problem in designing transportation networks is balancing the efficiency of transport and the cost of building and maintaining the network infrastructure. Putting this optimization problem in the context of ant transport networks, directly connecting all possible destinations would make transport within the colony as efficient as possible. However, that would also increase the costs and risks of maintaining an extensive trail network. Excavating tunnels is energetically demanding [10], while aboveground trails can still require maintenance [11] and may increase the risk of predation [12]. On the other hand, we rarely see ants minimizing the total trail length of their networks. Argentine ants (*Linepithema humile*) were shown to create networks that minimize the total length of trails, but only under laboratory conditions where ants were densely populated [13]. In the wild, networks of multiple species, including Argentine ants, balance cost and efficiency in network construction [8]. To understand how such networks can arise and persist, we should consider colony networks' resilience to perturbations.

Perturbations to ant transportation networks can manifest in the loss of trails, nests or stable food sources due to abiotic or biotic causes. In some cases, trails can become impassable, hindering the connectivity of the networks. Arboreal ants, which are restricted to moving along the branches of their home tree, often face the loss of trails as heavy wind or large-bodied animals can break off branches [14], whereas ant species moving on the ground do not face such strict topological constraints in placing their paths. Loss of nests can affect either type of network: nests can be lost at random, for example, due to weather-related events or being trampled by a large animal. In other cases, predators can target specific nests; for example, echidnas (*Tachyglossus aculeatus*) are more likely to predate larger Australian meat ant (*Iridomyrmex purpureus*) nests [12]. Ant species foraging on stable food sources incorporate those resources into their networks. Consequently, losing a key food resource can potentially alter their network structure. Australian meat ants [15] and wood ants [16] rely on honey-dew-secreting insects dwelling on trees for their carbohydrate intake. In many cases, only a few honey-dew-providing trees are responsible for supporting a colony of several populous nests [17], making efficient transport from trees to nests an important factor in nest establishment [15,18]. The loss of trees incorporated in the transportation networks can substantially change their structure [19]. Given that perturbations pose a frequent threat, we can expect ants to address the associated risks to maintain their networks in the long term.

There are two main ways to respond to perturbations: reactively or proactively. In the first case, colonies react to perturbations by making rapid changes to quickly restore network connectivity. Argentine ants can quickly establish pheromone trails [13] to restore connectivity, eliminating the need for robust topologies. Similarly, turtle ants (*Cephalotes goniodontus*) use a path-finding algorithm that is adaptable to varying tree topologies, allowing them to respond quickly to broken links in their established trail system [14]. Argentine ant colonies can spread effectively, making them invasive outside their natural range [20]. Turtle ants also defend scarce nesting resources but can quickly occupy new pre-existing cavities when they become available [21]. The ability to colonize new sites rapidly is key to their survival. In the second type of response to perturbation, ants proactively construct robust networks to withstand to withstand the effects of perturbations. This proactive strategy is common in species that build networks slowly. Wood ants build large nests where they can dwell for many years [18], and they rely on static food resources that are integral parts of their colony networks. Since both efficient transport and supply security are important for wood ants, their networks' structure balances cost and efficiency to improve the networks' ability to endure perturbations [8]. To fully understand the optimization of transportation networks facing perturbations, we must consider a third important measure alongside efficiency and cost: robustness, which is the networks' structural capacity to maintain function after perturbations [22].

Wood ant colonies make an ideal model for studying how environmental perturbations affect polydomous ant transportation networks. The foraging resources, trees, are naturally integrated into these networks, allowing us to observe how environmental changes directly influence network properties and the balance between cost and efficiency. Empirical perturbation of wood ant networks, by excluding the tree with the strongest foraging trail for a year, resulted in networks fragmenting into smaller components [19]. These colonies also began to forage on new sources, further changing their network structure in response to induced changes in resource distribution. While this study shows examples of network restructuring in response to one specific form of perturbation, to develop a comprehensive understanding of how wood ant transportation networks react to environmental disturbances, a range of perturbation types should be applied. Furthermore, each colony network has a unique structure that affects its response. This uniqueness makes it difficult to compare the effects of different types of perturbations across multiple networks. Thus, measuring the impact of a range of perturbation types would potentially involve so many colonies that it could exceed the limitations of empirical studies and risk damage to these keystone woodland species of conservation importance [23,24]. To extend and generalize the knowledge gained from the empirical studies, we have created a model to simulate the dynamic restructuring of wood ant networks.

Here, we present a comprehensive simulation model describing the perturbation response of wood ant colony networks as a model system for the impact of environmental perturbation on transportation networks. We based and validated our model on long-term observational data and experimental results from colony network perturbations. The simulations accurately capture the most important nest-level dynamics of colonies over three active seasons: changes in nest size and trail use, the establishment and abandonment of new nests and trails, and the employment and abandonment of food sources. With this model, we can test the effects of different perturbation treatments on the same networks, which would be impossible to do on natural colonies. Our goal with this empirically based simulation approach is to explore how well we can understand colony resilience to perturbations by applying rules derived from stable environments and limited perturbation data. We wanted to see whether these rules give the colonies the capacity to restructure effectively when the resource landscape abruptly changes. Based on the observation that the efficient access of valuable resources plays a significant role in structuring transportation networks like those of wood ants, we hypothesize that networks will exhibit not just an immediate but also a prolonged restructuring when their most heavily used resources are perturbed compared with random or weaker perturbations. Empirical manipulations [19] show that excluding a heavily used resource can decrease network efficiency by approximately 54% (95% confidence interval −96% to −10%, *n* = 4). Compared with this, we predict that excluding random or weakly used resources will lead to minimal changes and rapid restoration of network efficiency in network efficiency over the course of the simulation experiments. Furthermore, we hypothesize that the strength and duration of the perturbation will interact in determining the network’s response. We predict a substantial difference in network recovery between temporary and permanent removal of heavily used resources, with permanent loss leading to more persistent disruptions. By contrast, we expect no substantial effect of disturbance duration on recovery after the removal of weakly used resources.

## 2. Material and methods

### (a) Simulation model

**Figure 1.**
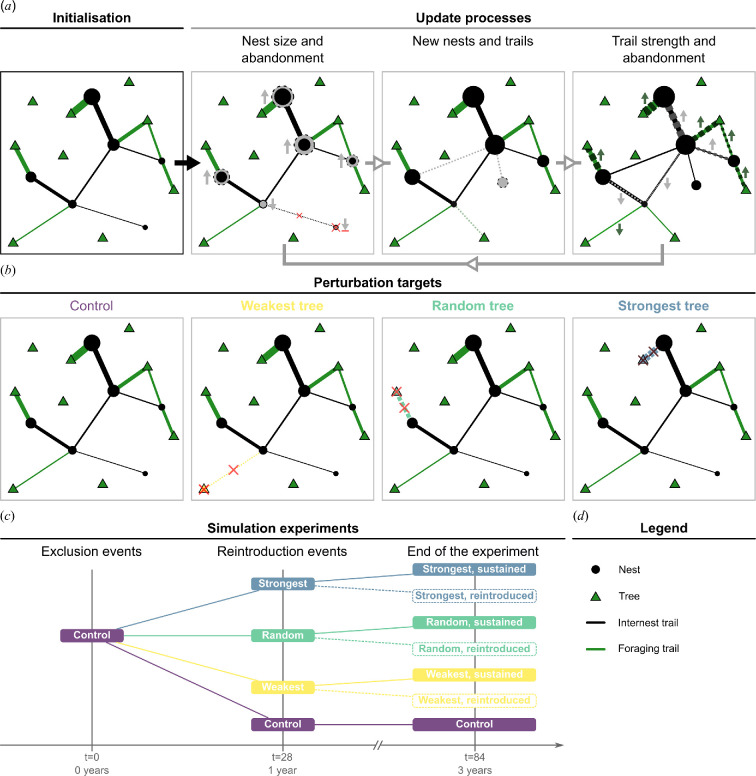
(*a*) Overview of network initialization and update processes, using an example network. (*b*) Illustration of different perturbation targets in the experiment. (*c*) Overview of the implementation of treatments in the simulation experiments. (*d*) A legend for the colony maps in the figure. (*a*) The model is initialized by generating a random network. Trees surround the nests that connect to other nests via internest trails or to trees via foraging trails. Nest size measures the number of ants inhabiting the nest, and trail strength is the density of foragers using the trails. The following processes update the network state in each iteration. Logistic growth updates the nest sizes based on the rate they gather resources and lose them to other nests. Nests shrinking below the threshold get abandoned. Next, nests can probabilistically bud new nests proportional to their size. New nests connect with an internest trail to their parent nest before all other nests are allowed to form new internest trails with other nests and foraging trails to trees. Lastly, an empirical formula updates trail strengths based on the nest sizes and the trail lengths. Trails weakening below the threshold get abandoned. (*b*) The different perturbations implemented on the same networks. In each network, the tree with the *strongest*, a *random* and the *weakest* cumulative foraging trail strengths are selected as focal trees for exclusion. No tree is excluded in the *control* treatment. (*c*) The simulation experiments start at *t* = 0 by excluding trees with the strongest and weakest cumulative foraging trail strengths and one at random in different copies of the network. Alongside the unperturbed control, the three versions run parallel for an entire active season to *t* = 28. Here, the focal trees are reintroduced to the networks in new separate versions. The simulation continues with the six treatments based on the perturbation target (weakest, random, strongest), type (sustained, reintroduced) and the control for two more active seasons. Timesteps *t* = 56 mark the end of the second, *t* = 84 the third season and the end of the experiments.

We provide a complete, detailed model description, following the ODD (overview, design concepts, details) protocol [25–27] in electronic supplementary material, S1. In general, the model aims to improve our understanding of the structural changes happening in multi-sink, multi-source transport networks in response to environmental perturbations. Specifically, we designed the model to accurately describe the structure, dynamics and perturbation responses of wood ant (*Formica lugubris*) colony networks. For this purpose, we parameterized and validated the model against empirical data (see §2c and electronic supplementary material, S1.3). See figure 1 for an overview of how the simulation and experiments work.

The model represents networks as graphs with three types of entities: ant nests (nodes), trees (nodes) and trails (edges). Each nest has a unique identifier and spatial coordinates. Nests have a size representing the number of ants inhabiting them. Nest status changes from the default *active* to *abandoned* if nest size drops below a threshold (see electronic supplementary material, table S1.3). Trees have the same state variables apart from size. Tree status can be set to abandoned to exclude the tree from the network and changed back to active to reintroduce it. Trails represent ants transporting food from trees to nests on foraging trails and between nests on internest trails. They are identified based on the nodes they connect, with their length measured as the Euclidean distance between those nodes (m). Trail strength is the density of ants using the trail (ants/mm). Trails are not allowed to cross, in line with empirical observations [18]. The networks can occupy a 60 × 60 m space, providing sufficient space for them to expand during the course of the experiments. The model is first initialized, then updated at 1 week intervals for three active seasons. An active season corresponds to the active foraging period of *Formica lugubris* in the UK, spanning seven months between April and October. The simulation starts with an initial burn-in period starting at *t* = −8. The first whole season starts at *t* = 0 with resource exclusion in the treatment groups, then these resources are reintroduced in the corresponding treatment groups at *t* = 28, the start of the second season (see figure 1*a*,*b*). The simulation stops at *t* = 84, the end of the third season (see figure 1*c*). We chose to model three active seasons to align with the timeframe for which we have sufficient empirical data for parameter estimation and validation, ensuring that our model accurately reflects the network dynamics directly influenced by the perturbations.

The model is initialized by generating networks using the morphogenesis model of Lecheval *et al*. [28] with modified tree placement mechanics (see figure 1*a*). First, the trees are randomly placed on the map with an inhibitory distance of 2 m at 0.01 trees per m^2^ density. The number of nests is drawn from an empirically fitted negative binomial distribution restricted to the empirically observed range of nest numbers (between 4 and 20). The nests are placed sequentially at random angles and distances drawn from an empirical gamma distribution, with distances below 1.5 m redrawn from a uniform distribution between 1.5 and 2. Nest sizes are drawn from an empirical gamma distribution but capped at the 95th percentile of empirical nest size distribution. Once all the nests are in place, each forms a single internest trail with another nest. Next, all nests draw their number of foraging trails from an empirical Poisson distribution. Nests get their foraging trails sequentially in reverse order of size, connecting to the closest available tree that is reachable without crossing existing trails.

After network initialization, the following main processes update the state variables at each timestep (see figure 1*a*). See electronic supplementary material, S1.1.7.1 for a detailed description of the processes. First, nest sizes are updated using a discrete logistic growth formula. The realized growth rate of each nest depends on its net food income. The net food income is calculated as the rate at which the nest gathers food from trees and other nests minus the rate at which other nests take away food from the nest. The nests grow when their net food income is positive and shrink if it is negative. See electronic supplementary material, S1.1.7.1.1 for a detailed description and the formulae. Next, nests with sizes below the fifth percentile of empirical nest sizes are abandoned along with all associated trails. After this, active nests can bud a new nest with a probability proportional to their size [18]. New nests are placed at a random distance and angle relative to the parent nest, using the same process that was used for nest placement during initialization. Each new nest is connected to the parent nest with an internest trail. Subsequently, all active nests can stochastically form new foraging and internest trails with a small constant probability. Lastly, the trail strengths are updated based on nest sizes and trail lengths using empirically parameterized gravity formulae fitted separately for foraging and internest trails (see electronic supplementary material, S1.2.3 for details). Gravity formulae are commonly used in transportation models to predict traffic flow between two locations based on population size [29].

We chose and implemented the above processes based on the following design concepts. While all patterns arise from the individual behaviour of ants, we can capture the most important structural changes in the network (abandonment and formation of nests and trails) at the nest level. We designed the model to describe the networks using the same state variables that were recorded in the field, allowing us to compare the two directly to validate the model and to be able to produce predictions that can be directly observed in the field. Wood ants can use their mounds for several years, forage on static resources, often returning to the same trails after dormancy [30]. Their networks change slowly during their active season [18]. We scheduled the model to precisely describe changes and allow the perturbations to show their effect (weekly updates over 3 years). Apart from generating the initial networks, stochasticity is only used for nest budding and the formation of new foraging and internest trails connecting nests. The literature describes the spatial position of newly budded wood ant nests as random [18,31]. Wood ants in nature form new trails through a recruitment mechanism with positive feedback after the spontaneous discoveries of scouting [16,28], which can be reasonably modelled as a stochastic process at the nest level. We chose the gravity model to model trail strengths as a widely implemented and proven way of describing network traffic [29]. We fitted the formulae to observed data to provide improved trail strength predictions.

### (b) Experiments

To test our main questions, we performed resource exclusion experiments on 10 000 simulated networks by excluding different trees either temporarily or permanently and then compared them with the unperturbed *control* version. We implemented two *treatments*: in the first treatment, the perturbation was *sustained* throughout, while in the other, the tree was *reintroduced* to the network after one season. These two treatments could affect three *targets*: the tree with the *strongest* or *weakest* cumulative foraging trail strengths (the sum of the strength of all foraging trails leading to the tree) or a *random* tree (see figure 1*b*).

In all treatments, the simulation runs on the initialized control networks for the burn-in period. The exclusion treatments are implemented at *t* = 0, the start of the first season. On three different copies of the network, the tree with the *strongest*, the *weakest* cumulative trail strength or a *random* tree is designated the focal tree and excluded from the network, meaning that its foraging trails are abandoned, and it cannot receive new ones. The simulation runs in parallel, in four different versions (*control*, *weakest*, *random* and *strongest*) for a full active season until *t* = 28. Here, each of the three resource exclusion treatments splits into two. In one of the two treatments, the excluded tree is *reintroduced* to the network, meaning that it is available for foraging, but the foraging trails are not automatically reformed. In the second treatment, the perturbation is sustained, and the focal trees remain unavailable for the colonies. From this point, the simulations run for two more active seasons (*t* = 84) in the seven different treatment groups (*control*, *weakest sustained*, *weakest reintroduced*, *random sustained*, *random reintroduced*, *strongest sustained* and *strongest reintroduced*; see figure 1*c*).

### (c) Empirical data and model validation

We used long-term data from nine wood ant colony networks used in an empirical perturbation experiment [19] and additional subsequent mapping data comprising a total of 38−184 nests per year. Data cover the period 2012−2021 at Longshaw Estate in the Peak District, UK (53°18′33″ N, 1°36′96″ E). Colonies were mapped following the protocol of Ellis & Robinson [31] in the late summer (July–August) at their peak activity. We documented all actively used nests and the trees they used for foraging. We used a mound-volume technique [32] to estimate the number of ants inhabiting the nest. We recorded all internest and foraging trails and measured the length of the trails. We calculated trail strength (ants/mm) from the length needed to count ten ants on the trail. We used the data prior to the start of the empirical perturbation experiment [19] (2012−2016) to estimate the distribution of the following model parameters: nests per colony, internest trail length, foraging trail length, nest size, number of foraging trails per nest (*Estimation data*). See electronic supplementary material, S1.2 for further details.

To verify the reliability of the model predictions, we validated our model on empirical data (see electronic supplementary material, S1.3 for further details). We validated the control simulations against the five control colonies observed during the empirical experiment between 2017 and 2021 (*Control validation data*). Additionally, we compared the simulated strongest reintroduction treatment with the four treated colonies in the same empirical study, which were similarly subject to a temporary strongly targeted resource removal (*Exclusion validation data*) [18]. The properties of the control and the perturbed simulations structurally and dynamically resemble real networks quantified by the measures used to characterize the networks (see §2d). The median values in our simulations closely approximate those of the empirical networks, although the simulations exhibit a wider range of values. Furthermore, a subset of our simulated networks demonstrates significantly different efficiency and robustness values compared with the empirical dataset. We believe that the larger number of simulated networks (*n* = 10 000) led to more variability and outliers than our empirical study of a smaller set (*n* = 9). While the number of network components of simulated networks closely resembled the validation data (see electronic supplementary material, figure S1.6), simulation networks gradually fragmented over time. The empirical data used for model validation and parameter estimation show a significant discrepancy in network fragmentedness. The number of network components in the *Estimation data* exhibits a narrower range than in the *Validation data*, which more closely resembles the simulation results. See electronic supplementary material, S1.3 for all details.

### (d) Network measures

We describe the most important properties of the networks with measures of efficiency, robustness and cost, along with several other metrics to capture a broader range of network characteristics. Transport is more efficient on shorter trails. Thus, to calculate network efficiency, we first gather the length of all the shortest possible routes between any two nodes. Next, we calculate the inverse of these distances to assign higher efficiency values to shorter paths. Finally, we average these values to get the network efficiency measure. Network efficiency expresses the expected food transfer efficiency of ants on a randomly chosen trail in the network [33]. Robustness is the average of the relative network efficiency loss when a single edge is removed. Robustness expresses the expected loss in the ants’ resource transfer efficiency when a randomly selected trail is disrupted [22]. Network cost is the sum of the physical length of all edges, expressing the cost of maintaining the trail network [34]. We also calculated these measures for the nest and internest trail subnetworks (i.e. excluding trees and foraging trails). Furthermore, we used additional measures to provide information on network functioning and ensure that the simulations capture all relevant aspects of the real networks. Specifically, we calculated the number of nests, used trees, internest and foraging trails; the ratios of trees, internest trails and foraging trails to nests, the number of network components (number of connected, disjunct subgraphs), normalized network cost (cost divided by colony population size) for entire networks and internest trails only, and colony population size. See electronic supplementary material, S1.1.4.8.1 for exact definitions.

### (e) Software and code availability

We used R 4.2.3 [35] and additional packages listed in electronic supplementary material, S1.4 to prepare this study. All code and data used for formulating, running and evaluating our simulations, as well as all simulated networks and results, are available online. The empirical data can be accessed on Dryad at https://doi.org/10.5061/dryad.g4f4qrfxn. The electronic supplementary materials (S1−2) are hosted on Zenodo at https://zenodo.org/records/10610579, alongside the software code at https://zenodo.org/records/10610577.

## 3. Results

**Figure 2.**
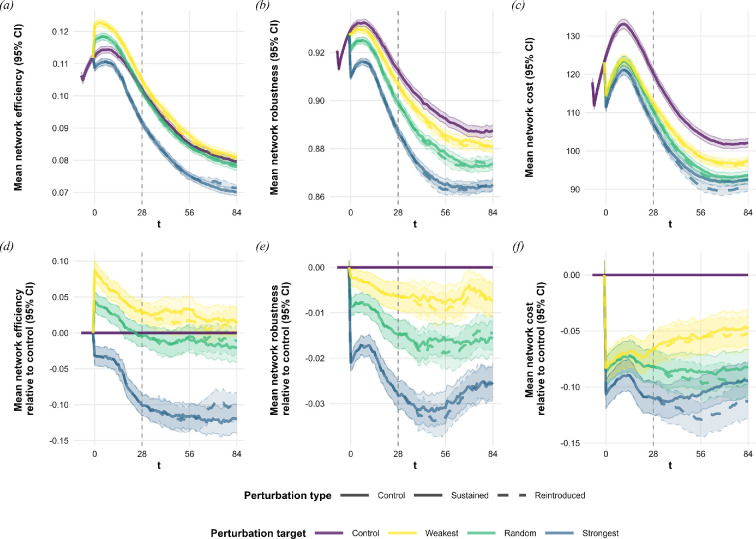
(*a*–*c*) Mean network measures with 95% confidence intervals and (*d*–*f*) mean relative differences from unperturbed controls (calculated by comparing each treatment instance to its unperturbed control instance of the same network) with 95% confidence intervals across different treatments implemented on 10 000 individual networks. Tree exclusions were implemented at *t* = 0 at the beginning of the first season. Trees were reintroduced in their respective treatment groups at *t* = 28 (vertical dashed line) at the start of the second season. Timesteps *t* = 56 and 84 mark the ends of the second and third seasons (see figure 1*c*). (*a,d*) Network efficiency increased in the *weakest* and *random* perturbation treatments but decreased in the *strongest* as a direct result of the tree exclusions. This initial impact increased in the *strongest* exclusion groups and diminished in the others over time. (*b,e*) The robustness of the networks was decreased by the perturbations in all treatment groups in line with the importance of the excluded tree and did not regenerate over the experiments. (*c,f*) Network costs decreased similarly due to the exclusions in the *weakest* and *random* groups and only slightly more in the *strongest*. Reintroducing the strongest excluded trees lowered the network costs modestly by the end of the second (*t* = 56) and third seasons (*t* = 84).

The tree exclusion treatments—excluding either the most strongly used, a random or the most weakly used tree—caused considerable adverse effects and restructuring in the networks. The exclusion treatments affected the networks’ efficiency in different directions depending on how the excluded tree was selected (figure 2*a*,*d*; table 1). The mean efficiency increased to 0.122 in the weakest and 0.117 in the random groups compared with 0.112 in the control group at the time of the intervention (*t* = 0). Meanwhile, the strongest perturbation treatment lowered the mean efficiency of the networks to 0.109. In the strongest perturbation group, examining the mean relative differences of the networks compared with their control versions (see figure 2*d*), we observed that the initial decrease in efficiency was followed by a brief period of stagnation. Then, between *t* = 14 and *t* = 28, there was a secondary drop in network efficiency in this treatment group. No such sharp changes were observed in the weakest and random perturbation target groups. By the end of the first season, the average efficiency of the random treatment did not substantially differ from the control, while the strongest treatment remained below. The reintroduction of the focal trees did not change the pattern of the weak and random exclusion groups: all remained similar to the control. However, the strongest exclusion group’s mean efficiency decreased compared with the control in both the sustained and reintroduced groups. By the end of the experiment, three seasons after the exclusions, networks were, on average, 11−12% less efficient in the strongest tree exclusion groups than the control. Examining only the internest networks, we see no difference between the treatment groups indicating that the treatments affected only the foraging and not the resource exchange efficiency of the networks (see electronic supplementary material, S2.10). The treatments also reduced mean robustness, with the decline being most pronounced for the removal of most heavily used trees, followed by a moderate decline for the randomly targeted trees, and the least reduction for the weakest trees (see figure 2*b*,*e*; table 1). While this effect was less substantial (2−3% less at most compared with the control), it was consistent across all treatments, with no apparent signs of recovery.

**Table 1.** Mean network measures measures and relative differences from control networks (%) across the different perturbation targets and types from 10 000 individual networks at highlighted timesteps (*t* = 0, the time of perturbations; *t* = 28, the time of tree reintroduction; and *t* = 56, 84, end of each of the two remaining seasons).

network measure	perturbation target	perturbation type	*t* = **0**	*t* = **28**	*t* = **56**	*t* = **84**
network efficiency	control		0.112	0.102	0.085	0.08
	weakest	sustained	0.122 (8.8%)	0.105 (2.7%)	0.088 (3.2%)	0.081 (1.3%)
		reintroduced	0.122 (8.8%)	0.105 (2.7%)	0.087 (1.7%)	0.081 (1.4%)
	random	sustained	0.117 (4.4%)	0.102 (−0.5%)	0.084 (−1%)	0.078 (−2.1%)
		reintroduced	0.117 (4.4%)	0.102 (−0.5%)	0.085 (−0.5%)	0.079 (−1.1%)
	strongest	sustained	0.109 (−3.1%)	0.092 (−10.1%)	0.075 (−11.7%)	0.07 (−12%)
		reintroduced	0.109 (−3.1%)	0.092 (−10.1%)	0.075 (−11.9%)	0.071 (−10.3%)
network robustness	control		0.929	0.913	0.893	0.888
	weakest	sustained	0.928 (−0.2%)	0.907 (−0.6%)	0.886 (−0.8%)	0.881 (−0.7%)
		reintroduced	0.928 (−0.2%)	0.907 (−0.6%)	0.884 (−0.9%)	0.881 (−0.7%)
	random	sustained	0.921 (−0.9%)	0.899 (−1.5%)	0.878 (−1.6%)	0.874 (−1.6%)
		reintroduced	0.921 (−0.9%)	0.899 (−1.5%)	0.876 (−1.9%)	0.875 (−1.5%)
	strongest	sustained	0.91 (−2.1%)	0.887 (−2.8%)	0.865 (−3.1%)	0.865 (−2.5%)
		reintroduced	0.91 (−2.1%)	0.887 (−2.8%)	0.863 (−3.3%)	0.866 (−2.5%)
network cost	control		124.813	120.256	104.328	102.129
	weakest	sustained	114.474 (−8.3%)	112.42 (−6.5%)	98.435 (−5.6%)	97.41 (−4.6%)
		reintroduced	114.474 (−8.3%)	112.42 (−6.5%)	98.432 (−5.7%)	96.826 (−5.2%)
	random	sustained	114.016 (−8.7%)	110.487 (−8.1%)	95.263 (−8.7%)	93.743 (−8.2%)
		reintroduced	114.016 (−8.7%)	110.487 (−8.1%)	94.373 (−9.5%)	92.715 (−9.2%)
	strongest	sustained	111.51 (−10.7%)	107.033 (−11%)	93.208 (−10.7%)	92.483 (−9.4%)
		reintroduced	111.51 (−10.7%)	107.033 (−11%)	90.88 (−12.9%)	90.598 (−11.3%)
number of nests	control		10.582	11.04	10.68	11.082
	weakest	sustained	10.582 (0%)	10.847 (−1.7%)	10.415 (−2.5%)	10.793 (−2.6%)
		reintroduced	10.582 (0%)	10.847 (−1.7%)	10.456 (−2.1%)	10.834 (−2.2%)
	random	sustained	10.582 (0%)	10.776 (−2.4%)	10.269 (−3.8%)	10.548 (−4.8%)
		reintroduced	10.582 (0%)	10.776 (−2.4%)	10.309 (−3.5%)	10.633 (−4.1%)
	strongest	sustained	10.582 (0%)	10.515 (−4.7%)	10.003 (−6.3%)	10.321 (−6.9%)
		reintroduced	10.582 (0%)	10.515 (−4.7%)	9.977 (−6.6%)	10.388 (−6.3%)
number of trees	control		5.779	6.05	6.128	6.301
	weakest	sustained	4.779 (−17.3%)	5.49 (−9.3%)	5.663 (−7.6%)	5.879 (−6.7%)
		reintroduced	4.779 (−17.3%)	5.49 (−9.3%)	5.756 (−6.1%)	5.995 (−4.9%)
	random	sustained	4.779 (−17.3%)	5.281 (−12.7%)	5.407 (−11.8%)	5.595 (−11.2%)
		reintroduced	4.779 (−17.3%)	5.281 (−12.7%)	5.549 (−9.4%)	5.782 (−8.2%)
	strongest	sustained	4.779 (−17.3%)	5.212 (−13.8%)	5.296 (−13.6%)	5.475 (−13.1%)
		reintroduced	4.779 (−17.3%)	5.212 (−13.8%)	5.499 (−10.3%)	5.758 (−8.6%)

Network cost decreased similarly in the three different target groups at the time of the intervention (*t* = 0; see figure 2*c*,*f*; table 1). On average, excluding the weakest tree lowered the total cost by 8%, the random tree by 9% and the strongest tree by 11% compared with the control group’s average of 124.8 m. The reintroduction of trees had minimal impact on the mean network cost in the weakest and random target treatments. In the strongest exclusion groups, it resulted in a modest reduction of the average cost by 2% compared with the sustained exclusion observed at the end of the second and third seasons (*t* = 56, 84). In comparison, the treatments hardly had any effect on the mean internest network cost (see electronic supplementary material, S2.14).

**Figure 3.**
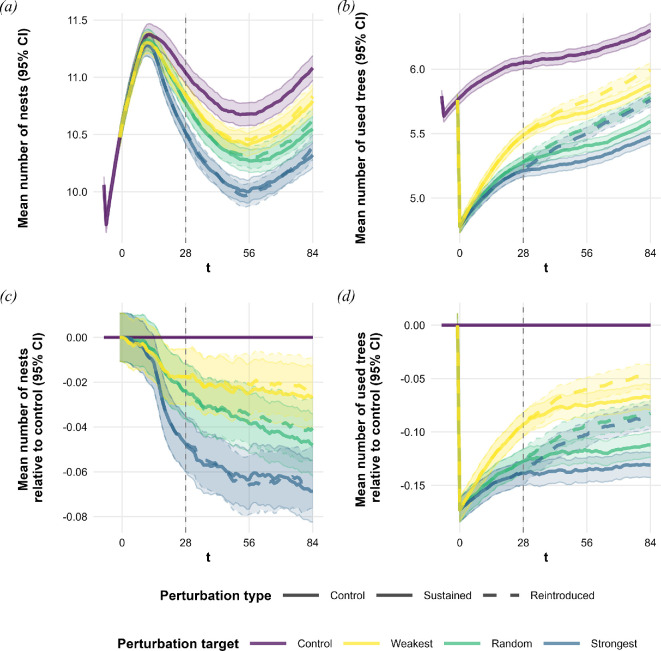
Mean number of network (*a*) nests and (*b*) trees with 95% confidence intervals, and (*c*,*d*) mean relative differences from unperturbed controls (calculated by comparing each treatment instance to its unperturbed control instance of the same network) with 95% confidence intervals, across different treatments implemented on 10 000 individual networks. Tree exclusions were implemented at *t* = 0 at the beginning of the first season. Trees were reintroduced in their respective treatment groups at *t* = 28 (vertical dashed line) at the start of the second season. Timesteps *t* = 56 and 84 mark the ends of the second and third seasons (see figure 1*c*). (*a,c*) Tree exclusions had a steadily developing negative impact on number of nests in the networks in proportional to the severity of the disruption. (*b,d*) The number of trees used by the networks regenerated faster after reintroducing the focal trees. In the *random* and *strongest* exclusion groups, reintroduction outweighed the effect caused by the differing importance of the targets.

The effect of the exclusion treatments on the mean number of nests in the colonies started to show by the end of the first season (*t* = 28; see figure 3*a*,*c*; table 1). In the strongest exclusion group, the colonies maintained 0.5 fewer nests compared with the control treatment’s 11. The other two target groups were affected less severely, showing an average decrease of 0.3 nests in the random and 0.2 nests in the weakest treatments. This effect was even stronger by the end of the second season (*t* = 56), with colonies maintaining 0.7 (strongest exclusion), 0.4 (random exclusion) or 0.3 (weakest exclusion) fewer nests. These differences still persisted after another season, three years after the exclusion (*t* = 84). Also, by this point, the reintroduction of trees reached a noticeable effect, with the reintroduction groups having, on average, 0.1 more nests than their sustained exclusion counterparts.

On average, networks in the treatment groups only slowly made up for the loss of their excluded tree (see figure 3*b*,*d*; table 1). One year after the perturbation events (*t* = 28), the weakest exclusion group used an average of 0.6 fewer trees than the control group (six trees), while the random and strongest exclusion groups used 0.8 fewer trees. Sustaining the exclusions allowed for only a slow recovery over the next two seasons. The weakest sustained exclusion group narrowed the gap with the control on average by 0.1 trees per season (−0.5 at *t* = 56, −0.4 at *t* = 84). The other sustained exclusion groups showed even slower recovery. At *t* = 84, the random exclusion group still used 0.7 fewer trees on average, while the strongest exclusion group used 0.8 fewer compared with the control from the first to the third year after the perturbation. Tree reintroductions, however, aided the recovery of the average number of trees used by the networks, most noticeably in the strongest and random exclusion groups. Both groups used, on average, 0.6 and 0.5 fewer trees than the control group at the end of the second and third seasons, that is still 0.1−0.3 more trees than used by their corresponding sustained exclusion groups. In this measure, the perturbation type had a larger effect than the target type. The boost in recovery was less pronounced in the weakest exclusion groups, but reintroducing the focal trees still allowed them to use an average of 0.1 more throughout the experiment.

We also see general processes in the simulated networks independent of the treatments. Control networks, on average, showed a brief increasing trend followed by a monotonic decline in efficiency, robustness and cost throughout the experiments. Network cost and robustness plateaued before the end of the experiments, while efficiency continued to decline (see figure 2; table 1). We observed a fluctuating pattern in the control networks' mean number of nests. The initial increasing trend reversed before the end of the first year. This decline reversed again around the end of the second year, and the average number of nests increased again (see figure 3*a*,*c*; table 1). The mean number of trees used by the control networks showed a monotonic increase throughout the experiment (see figure 3*b*,*d*; table 1).

Supplementary Material S2Electronic supplementary material, S2 offers additional, detailed results of all monitored network measures. It includes the number of internest and foraging trials, as well as the ratios of trees, internest trails and foraging trails to nests. It also contains results on the average number of network components as well as efficiency, robustness and cost measures calculated only for internest parts of the networks.

## 4. Discussion

Our results show that network dynamics suited to a static resource environment provide networks with only limited capacity to respond to resource perturbations. We find that in multi-source, multi-sink transportation networks, heavily used resources can act as structural organizers, playing a role that extends beyond simple provisioning. Even the temporary loss of these key resources can induce profound changes in network structure. However, contrary to our expectations, we did not observe substantial differences between temporary and permanent disturbances. Our findings suggest that self-organizing processes do not necessarily guarantee the restoration of the network to its pre-perturbation state or to an equally optimal configuration, even if disturbance is only temporary.

Heavily utilized resources are key structural organizers of network efficiency, and perturbing these resources triggers a different response and recovery compared with random or weak perturbations. Excluding weakly used or random sources had a contrasting effect on network efficiency, actually increasing efficiency, compared with the reduction in efficiency seen when targeting heavily utilized resources (figure 2*a*,*c*; table 1). This can be explained by the spatial organization and resource use within the networks. Wood ants rely heavily on nearby, well-connected resources while foraging at more distant trees with lower intensity [18,19,36]. Removing central, heavily used trees caused a considerable drop in efficiency due to the loss of short, highly efficient trails. By contrast, excluding weaker, more distant resources improved efficiency by eliminating longer, less effective trails. Even random exclusions showed a similar but less pronounced effect, underscoring that only a few key resources drive overall efficiency. Not only did we observe an immediate decrease in efficiency due to the removal of heavily utilized resources, but we also detected a secondary drop in network efficiency relative to the networks' control counterparts (see figure 2*d*). This trend appeared around *t* = 14, approximately half a season after the perturbation events took place. Beyond the initial impact, a delayed, non-trivial secondary decrease became evident. These observations suggest that the loss of heavily utilized resources not only disrupts the network initially but also triggers longer term restructuring processes that further reduce efficiency. Recovery patterns further support the organizing role of central resources. While networks subjected to random or weak perturbations regressed towards control efficiency, strongly perturbed networks did not recover (figure 2*a*,*c*; table 1). To better understand this, we inspected the number of trees used by the colonies. The reintroduction of excluded trees increased the number of trees utilized at a faster rate than in controls, showing that colonies re-incorporated them over two active seasons (figure 3*b*,*d*; table 1). However, despite this reintegration, network efficiency in the strong perturbation group remained impaired. These findings suggest that the self-organizing processes were unable to reincorporate valuable resources as efficiently as before the perturbations, highlighting the lasting impact of strong resource exclusions.

We hypothesized an interaction between perturbation strength and duration, predicting no substantial difference in recovery for weakly and randomly perturbed networks, while the reintroduction of heavily used trees would play a significant role. However, aside from the number of trees used by the networks (see figure 3*b*,*d*; table 1), we observed no apparent differences between networks exposed to temporary or permanent perturbations; both suffered from the same adverse restructuring. In our simulation model, which is largely based on data gathered in stable environments, there is no mechanism prompting colonies to preferentially re-engage with the reintroduced trees. Given that their primary food sources are typically stable, this assumption seems reasonable. However, without directed behaviour, colonies may not naturally reorganize to their previous state. This poses a challenge for the colonies, as both the efficiency and robustness results show that resource exclusions promote adverse restructuring even when resources are later reintroduced. We see that the simulated networks, produced by dynamic processes based on long-term empirical data, could not build their robustness back throughout the experiment, regardless of whether the resource was later reintroduced or not. Diminution of robustness (defined by the average relative network efficiency following the loss of a trail) means that colonies are persistently more vulnerable to future disturbances. As the decrease in robustness is in line with the intensity of the disturbance, we see again that this unfavourable restructuring is primarily driven by the loss of heavily utilized trees in the network. If a colony had an equally good alternative to the lost resource, they would have already utilized it. In this case, an appropriate response to resource loss would be to reposition the nests, which takes time and comes with substantial costs [18]. Newly founded nests, while they are still small, are also more vulnerable than well-developed nests, further decreasing the resilience of the colony network [18]. These results show that short-term restructuring has limited capabilities for responding to environmental perturbations when the network has gradually developed around static resources.

Our results have practical implications for populations of polydomous ant colonies and their conservation. Despite the adverse structural changes observed in their networks, the number of nests and trees used by the colonies showed only a moderate decrease. Similarly to the real-life experiment, we found that, on average, excluding a single resource from the network did not exceed the colonies’ resilience or regenerative capacity [19]. However, we should take these findings cautiously, as all networks are unique. The perturbations' negative effect on the number of nests and trees is still a cause for concern. As wood ants are often the subject of conservation efforts [24], we advise stakeholders to consider each polydomous wood ant an interconnected entity and focus on protecting heavily foraged trees, especially if the associated colony is small or they expect other environmental perturbations in the short or medium term [37]. Our results suggest that wood ants not only dwell but can only flourish under relatively stable environmental conditions.

Our simulated networks also displayed natural, developmental patterns over time, which were consistent across all conditions, including the control. These patterns can be attributed to the networks interacting with and adapting to their surroundings. While an extensive population of colonies at overlapping developmental stages would be expected to reach a stable state over time, individual colonies follow their own life histories, which can include restructuring, expansion, regression or deterioration. The observed general trends can be seen as a typical colony life history trajectory over the course of three active seasons, averaged across many individual colonies. The fluctuating pattern in nest numbers may result from an initial emergence of newly budded nests, followed by the decline of many as they struggle to establish themselves. Subsequently, a new set of budding nests may replace those that did not persist. The plateauing of network cost and robustness could be a part of the generated networks settling in the resource landscape. While the burn-in phase accounted for immediate instabilities after starting the dynamic simulation (see figures before *t* = 0), a long-term stabilization as a product of the modelled dynamics also happened. The overall trend in the decrease in efficiency could be related to the trail formation processes used in the simulation and the fragmentation of the networks (see §2c).

Our results show increasing network fragmentation under all conditions, including our control. Although real-life wood ant networks do exhibit fragmentation in response to resource removal [19], our empirical data suggest that natural colony networks maintain cohesion more effectively than our simulated ones (see electronic supplementary material, figure S1.6). While fragmentation is observed on the scale of whole networks, colony-level cohesion arises from the formation of individual trails. Wood ant nests typically connect to their nearest neighbours, but networks also feature long-distance connections [8]. The formation of long-distance trails is probably driven by the ability of high-quality resources to attract traffic from nests farther away compared with low-quality resources [28], so high-quality resources may play a pivotal role in shaping network robustness. In our model, we assumed that all trees have equal quality because we currently lack empirical studies on the quality of trees as food sources, how ants in nature react to this quality and how to compare it with the quality of nests that are also treated as food sources by the ants [38]. Empirical studies of food resource quality could further improve the predictive power of our models and help us better understand the role that high-quality, heavily used resources play in the development and maintenance of transport networks..

Despite sharing similar biological traits such as polydomous colony structure and tree-based foraging, Australian meat ants and wood ants exhibit distinct network optimizations. Meat ants prioritize cost-efficient food transfer, enhancing efficiency but compromising network robustness [39]. Unlike wood ants [8], efficient food transfer from the trees to the nests is seemingly more important for meat ants. Meat ants deliver honeydew from a tree to the closest nest [15]. They also preferentially establish new nests close to foraging trees [15], something we see no evidence for in wood ants [18,31]. This efficiency probably stems from the varying reliability of food sources observed in meat ant-utilized trees [15]. Polydomous meat ant colonies may handle the spatio-temporal variability of food availability by transporting it to the nearest nest and efficiently disseminating it within the colony [15]. The meat ants' increased reliance on internest redistribution compared with foraging trails could potentially mean the adverse effects of losing a resource are also more evenly distributed within the colony, generating less of an impact on directly affected nests than in wood ant networks. The differences between these two species in resource redistribution behaviour and the resulting network structure might lead to very different perturbation responses, highlighting the value of studying network resilience across a variety of self-organizing processes.

We can gain further insight into the overall perturbation response of the simulated wood ant colony networks if we compare our results with other popular network models. Scale-free networks have been widely applied to describe the structure of protein networks, the World Wide Web or social interactions [40]. Scale-free networks developing from a rule of preferential attachment generate important hubs in the network [41] making them weak to targeted perturbations affecting these hubs [42]. In random networks, on the other hand, there is no substantial difference between targeted and random attacks [42]. Ecological interactions, particularly trophic relationships, are also frequently studied from a network perspective. Studies on food webs provide valuable insights into how ecological networks respond to external perturbations. In natural settings, terrestrial and freshwater food webs typically exhibit scale-free structures [43]. However, the impact of human-induced disturbances often targets species acting as central hubs within these networks. Consequently, in regions experiencing increased anthropogenic perturbations, food webs have shifted structurally towards a predominantly random configuration, allowing them to withstand disturbances affecting these pivotal 'hub species' [43]. Our study shows that while self-organized processes using only local information can create efficient yet resilient transportation networks, these can still be particularly sensitive to strong, targeted perturbations, with potential long-term consequences even if the perturbations are reversed. Our approach highlights the importance of considering the dynamic response of networks to perturbations of different types as a key element structuring biological networks across levels of organization.

## Data Availability

All code and data used for formulating, running and evaluating our simulations, preparing electronic supplementary materials S1–2, as well as all simulated networks and results, are available on Dryad [44]. Supplementary material is available online [45].
